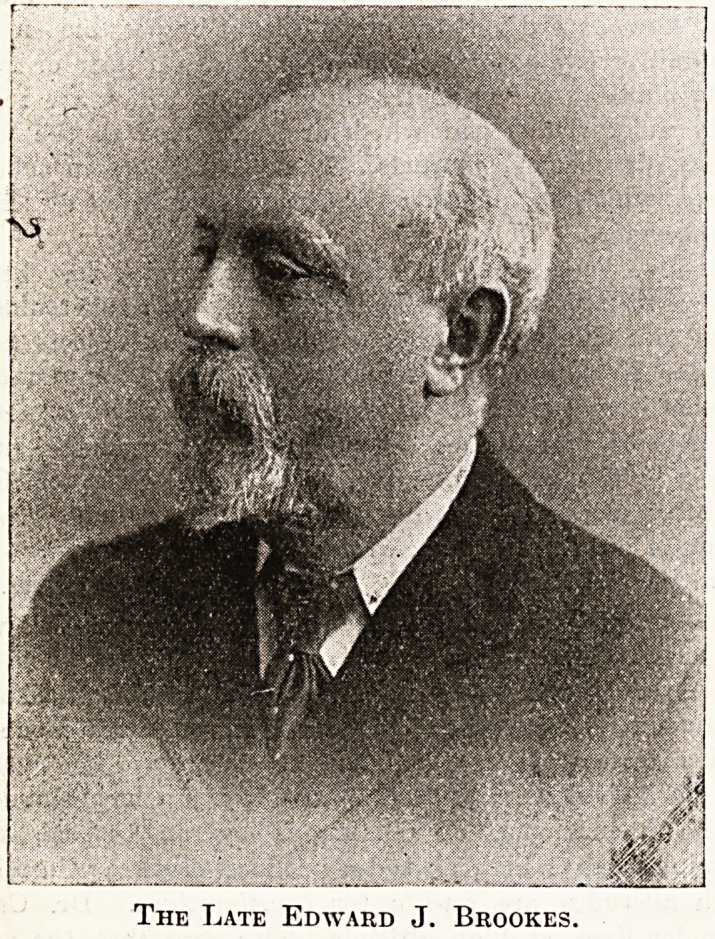# An Able Provincial Chairman

**Published:** 1913-03-15

**Authors:** 


### An Able Provincial Chairman.

### THE LATE MR. EDWARD BROOKES.

The Hospital Service loses an able administrator and a generous and enthusiastic supporter by the death of Mr. Edward J. Brookes, J.P., which occurred at his residence at Sutton Coldfield on the 25th inst., after a painful illness.

Mr. Brookes was associated with the management board of the Walsall and District Hospital for a period of forty-two years. He was appointed treasurer in 1882, and continued in that office until 1890, when he accepted the position of chairman. Under his guidance the work of the institution prospered and expanded; many important extensions of the buildings, and improvements in their technique, in which he was actively interested, were effected in the course of the eighteen years during which

The Late Edward J. Brookes.

he held the chairmanship. Amongst these were the partial rebuilding and the extension of the hospital in 1895, after the wrecking of two wards by a disastrous gale which swept over the Midlands on March 24 of that year; the erection of a new and up-to-date out-patient department and an excellent home for the accommodation of the nursing staff in 1902; a new operating theatre in 1903; the remodelling of a large portion of the buildings, the installation of a new system of heating and ventilation, and the erection of two new wards in 1906 and 1907.

Mr. Brookes was on terms of intimate friendship with the late Sister Dora, who was matron of the hospital when he first joined the board, and their official association was marked by earnest and strenuous efforts to promote the work of the hospital, a task which, owing to the peculiarity of prevailing circumstances, was by no means easy.

At the close of 1907 Mr. Brookes, though still an active man, declined to accept re-election as chairman, because he felt that the increasing needs of the institution and the importance of keeping pace with the times demanded the energies of a younger man. Only those who knew him intimately fully recognised the sacrifice he made to what he considered was his duty to the institution he loved when he firmly refused to reconsider his decision, and when a few years later he also relinquished his seat on the hospital board.

His association with the hospital, as well as with other local philanthropic institutions, was a record of personal service and generosity, and his genial personality won for him the affection of all with whom he came into contact.

## Figures and Tables

**Figure f1:**